# Users’ views on the use of a smartwatch app to collect daily symptom data in individuals with multiple long-term conditions (Multimorbidity): A qualitative study

**DOI:** 10.1177/26335565231220202

**Published:** 2024-01-10

**Authors:** Cassandra Kenning, Peter Bower, Nicola Small, Syed Mustafa Ali, Benjamin Brown, Katherine Dempsey, Elaine Mackey, Brian McMillan, Caroline Sanders, Ilina Serafimova, Sabine N Van der Veer, William G Dixon, John McBeth

**Affiliations:** 1 Centre for Primary Care and Health Services Research, Faculty of Biology, Medicine and Health, 12203 The University of Manchester, Manchester, UK; 2 Centre for Epidemiology Versus Arthritis, Manchester Academic Health Sciences Centre, 5292 University of Manchester, Manchester, UK; 3 Centre for Health Informatics, Division of Informatics, Imaging and Data Sciences, Manchester Academic Health Sciences Centre, 523398 University of Manchester, Manchester, UK

**Keywords:** Multimorbidity, smartwatch, patient-generated health data, user engagement, qualitative

## Abstract

**Introduction:**

Long-term conditions are a major burden on health systems. One way to facilitate more research and better clinical care among patients with long-term conditions is to collect accurate data on their daily symptoms (patient-generated health data) using wearable technologies. Whilst evidence is growing for the use of wearable technologies in single conditions, there is less evidence of the utility of frequent symptom tracking in those who have more than one condition.

**Aims:**

To explore patient views of the acceptability of collecting daily patient-generated health data for three months using a smartwatch app.

**Methods:**

Watch Your Steps was a longitudinal study which recruited 53 patients to track over 20 symptoms per day for a 90-day period using a study app on smartwatches. Semi-structured interviews were conducted with a sub-sample of 20 participants to explore their experience of engaging with the app.

**Results:**

In a population of older people with multimorbidity, patients were willing and able to engage with a patient-generated health data app on a smartwatch. It was suggested that to maintain engagement over a longer period, more ‘real-time’ feedback from the app should be available. Participants did not seem to consider the management of more than one condition to be a factor in either engagement or use of the app, but the presence of severe or chronic pain was at times a barrier.

**Conclusion:**

This study has provided preliminary evidence that multimorbidity was not a major barrier to engagement with patient-generated health data via a smartwatch symptom tracking app.

## Introduction

Long-term conditions (LTCs) affect one in four people in the UK, often requiring life-long management, and accounting for 70% of the health service budget.^1,2^ Additionally the number of people suffering from multiple LTCs (multimorbidity - MLTC-M) is rising globally. In the UK, around two-thirds of people aged 65+ are thought to have MLTC-M.^3^ Patients with MLTC-M face particular challenges in managing their conditions and interacting effectively with services, and often report poor outcomes.^4,5^

The Academy of Medical Sciences identified a number of research priorities to meet the global health challenge of MLTC-M.^6^ These include exploring the potential use of consumer technology (such as smartphones and wearables) to monitor symptoms of people living with MLTC-M (so-called *patient-generated health data* - PGHD).

When integrated into patients’ daily lives, smartwatches provide an opportunity to collect both regular symptom data and continuous objective measurements via sensors using a single wrist-worn device. This provides a potential foundation for (a) self-management through symptom tracking (b) supporting clinical care by integrating PGHD into clinical systems and (c) population health research by measuring changing disease states over time, complementing existing data sources such as health records.^7-10^ It is the third function that is the focus of the current paper.

Previous literature shows that patients with particular LTCs are willing to collect PGHD.^11^ However, people with MLTC-M face particular challenges. MLTC-M requires monitoring of more symptoms (and greater burden of data entry) and people may have reduced capacity for engagement due to the complexity of their health problems (‘disease burden’) and their extensive interactions with services (‘treatment burden’). It is still not clear if the outcomes from studies which focus on single LTCs can be applied in MLTC-M.

Watch Your Steps was a feasibility study to co-design a system for collecting PGHD in MLTC-M, and to examine the feasibility and acceptability to patients of collection using a smartwatch app for research. The quantitative results have been reported.^12^ This paper explores patient views of the acceptability of collecting daily PGHD for three months using the smartwatch app, and identifying barriers to and facilitators of ongoing engagement.

## Methods

### Study design

Watch Your Steps study was a mixed methods feasibility study, and is described in more detail in the main paper.^12^ The current paper reports the results of the qualitative study.

### Study population

Up to 60 patients aged 18+, with at least two clinician-diagnosed LTCs were eligible to participate, with a sub-sample of 20 selected for interview. Due to our interest in tracking physical activity, bedbound or housebound participants and those who lacked the capacity to provide informed consent were excluded.

### Participant recruitment

All participants recruited to the trial were asked for consent to be contacted to take part in an interview about their expectations and experiences using the smartwatch app. During the recruitment process participants were made aware that only a sub-sample of up to 20 participants would be contacted for interview. All participants consented to be contacted, those selected for interview were contacted again prior to interview to ensure they were still interested in taking part. The aim to interview 20 participants formed part of the original study protocol and was thought to be sufficient to explore the range of issues around app usage.

Participants were selected to ensure they represented a range of characteristics including age, gender, ethnicity and number of LTCs. We did not purposefully select those with higher levels of engagement and the interviewer did not know what the levels of engagement were for each participant at the time of interview. We compared demographic and usage characteristics of the subsample with all participants after participation, to ensure they were similar.

### Aims

The aim of the interviews was to enable insights into the feasibility and acceptability of data collection through the smartwatch in a sample of patients with MLTC-M, and to help identify barriers and facilitators of ongoing engagement.

### Interviews

Interviews were thought to be the most appropriate method for collecting data as we wanted to collect each participant’s personal experiences of using the app to collect PGHD. To facilitate this, a semi-structured interview schedule was developed to ensure key areas were discussed. Box 1, below details key topic areas. The process was iterative, and all participants were asked if there was anything further they wanted to raise that had not already been covered. This allowed for the interview schedule to be amended where needed to include new themes or to explore issues in more detail.

Baseline interviews were conducted face-to-face with 20 participants between Dec 2019-May 2020. End-of-study interviews were carried out at three months (post onboarding) with the same sub-sample. Fifteen interviews were completed remotely via telephone, two via Zoom, and one was completed face-to-face, with a total of 18 end-of study interviews completed between Feb-July 2020. An encrypted audio recorder and earpiece adaptor were used to record telephone and/ or Zoom interviews, a USB connector was used to securely transfer the data. All onboarding events and interviews were conducted by an experienced qualitative research associate, NS (female), using semi-structured interview schedules. The interviewer had no prior relationship to participants prior to study commencement.

Box 1- Topic areas for interviews

The areas for interview at the end of the study included:

• How their health had been since they last met the interviewer
• Participants’ experiences of responding to questions in the Watch Your Steps app
• Whether condition specific questions were appropriate to their LTCs
• Participants’ views about the data collected in the background by the watch sensors
• Whether tracking participants symptoms and activities was useful
• Any technical or other issues the participant found while using the app
• Participants’ views on using this device/app over the longer term.

### The WYS app

The app was pre-loaded onto Fossil Sport smartwatches, with other smartwatch apps disabled to optimise battery life. Participants were asked to select from six disease areas, all those which were applicable. The disease areas were (a) bone, joint or muscle (b) skin (c) heart or lung (d) stomach or bowel (e) kidney and (f) mental health. Each area had organ-specific symptom questions, asked at varying intervals throughout the day, alongside generic health questions (single questions each on: sleep, wellbeing, pain, mood, fatigue, stress and function). Active tasks included: ‘Sit stand test’ (Sit down in a chair, fold your arms across your chest, then stand up and sit down twice); ‘Walk, turn and return test’ (Walk ten steps forwards, turn around, and walk ten steps back’); and ‘Tap test’ (How many times you can tap the screen in 5 seconds).

Five times a day (8am, 12pm, 4pm, 6pm, 8pm), the app provided an alert about question sets due for completion. In addition, participants were able to answer any of the questions at any time point if they wished to record a particular symptom. An active task request showed on the watch once per day on three days per week. It was anticipated that total time for completion of question sets and active tasks would not exceed five minutes per day, spread at intervals. For full details, see the main paper.^12^

Data were collected seven days a week for 90 days. This time period was thought to be long enough to see variation in symptoms, and a reasonable test of endurance and sustained engagement. There was no feedback of data during that period due to resource constraints in the development of the smartwatch app.

### Qualitative analysis

Audio-recorded interviews were transcribed verbatim. The anonymised transcripts were then imported into Nvivo 12 pro^13^ for analysis. CK conducted thematic analysis drawing on techniques of a grounded theory approach including open coding to develop initial themes, and an exploration of relationships between themes and across cases using constant comparison. A number of *a priori* themes were identified (see box 1) as key to understanding acceptability in this population. Within those general themes, the categories or sub-themes were identified inductively as emergent from the data. A map of themes was created to help draw links between sub-themes and to develop the overarching themes. CK met regularly with the wider team to discuss the analysis.

To further explore differences in engagement found in the quantitative study we looked at whether the levels of engagement and lived experience of using the Watch Your Steps app were the same for all participants. We wanted to see if there were differences for older participants or those with a greater number of LTCs. To explore this, participants were split into age groups <60 (n=13) vs ≥60 (n=7), and <3 LTCs (n=15) vs ≥3 LTCs (n=5), and comparative analysis conducted to see if either of these factors influenced engagement or experience.

## Ethical approval

The study received a favourable NHS REC opinion and HRA approval (19/WM/0307).

## Results

In total, 62 people were screened for eligibility, 53 (85%) were onboarded and 20 completed baseline interviews. Eighteen follow-up interviews were completed (one face-to-face, the rest telephone/online) and used in this analysis as these provided participant experiences and feedback on using the system. Follow-up interviews were conducted between February-July 2020, and ranged from 27 to 70 minutes (mean 50 minutes).

The demography of the interview sample was similar to the total sample except for gender and engagement. Female participants made up half (n=26) of the total sample but only 7 interviewees (35%). Those who were interviewed had a higher overall response rate compared to the total sample, with the overall group averaging 54.0 correct responses compared to 61.4 for the interview sample (Participants had a 2 hour window to complete their responses following the prompt on the app, to be considered 'correct completion'). Table 1

**Table 1.** Demographic details of interviewed sub-sample.

Characteristic	Categories	Number (%)
Age group	18-29	2 (10)
	30-39	2 (10)
	40-49	3 (15)
	50-59	6 (30)
	60-69	4 (20)
	70-79	3 (15)
Gender	Male	13 (65)
	Female	7 (35)
Ethnicity	White	17 (85)
	Mixed or non-white	3 (15)
Number of disease areas^a^	1	7 (35)
	2	8 (40)
	3	2 (10)
	4	2 (10)
	5+	1 (5)

^a^All participants were confirmed as having two or more LTCs during eligibility screening. The number of pre-specified disease areas refers only to the specific, named organ systems listed above.

### Engagement with WYS App

To explore possible barriers to engagement, we looked at how easily participants were able to fit the data collection process into their daily routine. The majority reported finding the watch easy to use and to develop a routine for using it:

“Because after the initial first week or so it just became so normal for it to be there, it just became part of the routine. And I didn’t really then give it much thought” (9658, M, 50-59, 2 conditions)

Physical obstacles (such as pain) which are discussed later, were the only real barriers reported to impact some participants ability to complete tasks. However, this was not reported to be a direct result of the apps useability or participants willingness to use it. To further explore engagement, participants were asked to think about the number of alerts they were receiving each day and the number of questions or tasks within each of those alerts, to gage the level of burden participants may have felt.

### Number of alerts per day

Participants received five alerts, seven days a week. The majority were happy with the number of alerts, accepting the need to collect sufficient data. The time intervals were also generally thought to be convenient. However, a small number reported that the timings did not fit in with their daily routines. They suggested that this might be something people could personalise at set up, selecting what times would be convenient for them:

“Yeah. It did it several times a day, I think it's what is it, eight o'clock, twelve o'clock and four, something, and then again eight at night, but they're all convenient times when you're actually breaking from what you're doing.” (5262, M, 60-69, 4 conditions)

Not everyone thought the number of alerts was appropriate. For example, one older participant reported frustration at having to answer the same questions each time. However, it is possible that this may be due to repetitiveness of the questions rather than the frequency of the task:

“I found it frustrating having to answer questions so frequently. It seemed like one would come up and I'd think to myself I only answered that like half an hour ago. In reality it was probably two hours, but the same question, ‘How is your pain at the moment’ or ‘How are your stress levels’ or whatever.” (5454, M, 60-69, 4 conditions)

Conversely another participant commented on the fact that they had expected more prompts and more questions (however this person withdrew after just 7 days due to a skin reaction to the watch):

“I think I expected a bit more involved rather than just a few simple questions four times a day over 16 hours.” (4489, M, 50-59, 1 condition)

Whilst the majority were happy with the frequency of the alerts, personalisation that allows participants to tailor the number and timings of the alerts each day may improve long-term use for others.

### Number of questions/tasks per alert

Each participant received a different number of questions within those tasks according to their LTCs (for example, someone with joint and heart conditions would on Monday answer 14 ‘generic’ questions, one active test, plus 7 organ-specific questions, a total of 22 tasks). Most participants reported that they were happy with the number of questions/tasks but some noted they had expected more in a research study, to collect as much data as possible:

“Just the way the alarm went off and you do your three or four [questions] you think, well, you know, is that it?” (4489, M, 50-59, 1 condition)

However, it is noted that these participants only selected one disease area to monitor, and thus had a lower number of questions than some other participants. Overall, participants did not seem to find the level of input required burdensome:

“And I didn’t find them intrusive at all. Once you get into the habit of doing them you become used to doing them bang, bang, bang, dead easy. I found it really simple to do. It wasn’t a bind. I didn’t feel as though it was a chore at all”. (9658, M, 50-59, 2 conditions)

#### Appropriateness and accuracy of the questions

The majority reported that they found the condition-specific questions appropriate to their LTCs and no one suggested any specific questions that were missing:

“It covered my symptoms and my problems well. Whether that would be the case for others I can’t comment because I don’t know what other people’s issues would be. But for my symptoms and my problems I thought it was good.” (9658, M, 50-59, 2 conditions)

However, there were a few participants who felt that rather than adding other questions, it would be useful if you could remove some symptom questions which, although often associated with a condition, they might not experience themselves. For example, one participant had psoriasis but did not experience itching and so was having to input ‘0’ several times a day which caused annoyance as they felt no useful data would come from it:

“Yeah, they were all perfectly okay. But there's one that…I mean I answered it, but it was all about itch. I don't have any itch, so it was an irrelevant information thing, because you're always going to get what was the daily itch, not bad, what was the average itch, not bad. It's a nonsense, I don't know why I got that question.” (5262, M, 60-69, 4 conditions)

Another participant expressed irritation at having to respond about bowel movements when they did not feel they had any problems in this area, despite their stomach condition:

“Well, the only other thing that I think, it should be a bit more tailored to the individual, instead of all these sort of spurious ones, like bowel movements. So, if you have no real problems, underlying problems with your digestive system or anything like that, I can’t really see the need to [answer that]...” (8010, M, 70-79, 2 conditions)

There was some concern that the questions were too general and they were unable to specify whether a symptom was related to one or another of their conditions. Some felt that without any specificity or ability to clarify answers via free text, the technology might not capture an accurate reflection of someone’s condition.

“Well, from a personal point of view, if it was a bit more specific perhaps to your actual joints. Perhaps it should’ve said, ‘are your joints stiff’ or are your whatever stiff today or, you know, that sort of thing. There is a general one, stiffness, but that’s, if I recall correctly, it’s to do with sort of getting out of bed in the morning, how stiff you were, whether you could get out of bed without falling on the floor and that sort of thing. But for a specific illness such as arthritis, it might be better to aim at, you know, the sort of main joints that you would expect people to struggle with.” (8010, M, 70-79, 2 conditions)

Another participant discussed how it is not always enough to record one factor of a symptom:

“Well, if I've had a flare up and the flare up had been itching I would have reflected that in the scale that the itch had. But what I couldn't reflect was any changes in the look of the rash, because that's very specific, I would think, it's in my right hand or my left leg, is that too specific to be able to do, I don't know.” (6578, F, 60-69, 1 condition)

Participants had high expectations for the specificity of symptom questions. Whilst it was reported there was nothing specific missing from the questions, again participants wanted the ability to tailor or remove questions relating to symptoms they did not personally experience. The ability to clarify or expand on some symptoms was also thought to be more useful for some participants.

#### Data collection period and long-term use

We also queried participants on the three month duration of data collection. Most reported that they did do this with the exception of one participant who had to stop wearing it after one week due to skin irritation. Some were happy that the three months was sufficient to give an accurate picture of their symptoms:

“I think three months should be okay, just for myself, three months is okay, is the right timeframe to answer those questions.” (8921, F, 40-49, 1 condition)

However, a few did reflect that they experienced seasonal differences in symptom levels or that ‘flare-ups’ of symptoms could occur at random times and that this had not been captured in the 3 months. Measuring longer term changes with a view to remission of some conditions was identified as a key factor for some:

“With a condition like mine I can be stable for three, four, five, six months, no problems, and then all of a sudden crash. I was saying I’m always three days away from being in hospital, because I can crash that quickly. If I go downhill it’s like overnight it can be. For my condition I think it would have to be 12 months minimum.” (9658, M, 50-59, 2 conditions)

A small number of participants reflected that they were happy to complete the study but would not want to use the app for a prolonged period. Another participant linked the lack of ‘real time’ feedback to the length of time they would be willing to wear the watch:

“Definitely…it was fine, I was starting to get really bored with it after 90 days. If you were getting more out of it, I’d be more happy to keep going.” (4987, M, 50-59, 2 conditions)

This indicates a tension between the need for long-term use to accurately reflect changes in LTCs with the burden that is associated with that. Increased interactivity and feedback from the app may help to engage users for longer periods.

#### Barriers to engagement

Three factors were raised as barriers to engagement in both the short and longer term: (a) repetitiveness of the questions, answers and scores they were inputting (b) that the active tasks were too easy (c) that there was no immediate feedback meaning that it could not be used for self-monitoring.

### Repetitiveness

A key issue for research is whether people will stay engaged and actively input data even when their symptoms are stable. The repetitive nature of the answers during this time can be a barrier, as participants need to understand (and research teams need to be clear) that to create an accurate reflection of their daily symptoms there does not need to be variety in their responses. This was evident in some cases:

“Yes, I just felt like, you know, not to sound harsh but answering the same questions just did feel a little bit tedious. I was there for a while so it made me feel like sometimes not wanting to answer the questions.” (1224, F, 18-29, 1 condition)

Some participants were concerned that lack of variation in their responses would not provide the research team with ‘interesting’ data.

“Not really. You don't do it that many times a day that it's intrusive. It's me thinking about what it was, the value of what I was doing, well, you're not giving them any variation here. But there again you're not supposed to invent variation either, are you?” (5262, M, 60-69, 4 conditions)

However, for many, the fact that they were taking part in research meant that they remained engaged:

“I think I was coming from an angle where I’m conscious that this is a study so, it's just part of the study. So I’m not going to get bored of them because I want to give you the best information as I can. But I can understand why people have said that that you knew what questions were going to come up. But I don’t think that changed how I answered any of the questions.” (8778, F, 30-39, 2 conditions)

### Active tasks

Most participants reported completing the active tasks regularly at the beginning but that it reduced over 12 weeks. Of the three active tasks, the ‘tap test’ (which was novel to the app and gave immediate feedback in terms of a score) was enjoyed most. The ‘sit stand test’ and ‘walk, turn and return test’ were perceived to be more difficult because they were not always convenient:

“They were fine. I think the tap test, I always did that. The walking one I sometimes found difficult because of where I was and what I was doing because it wasn’t always appropriate for me to do that but when it was I did do it.” (8778, F, 30-39, 2 conditions)

Other participants expressed uncertainty around the purpose of the active tests:

“Yeah. I think it’s a similar thing to the data, I didn’t know the reason or it was hard to know what I was supposed to be doing. ‘Your best time on the sit, stand was one second’. So does that mean it was being used as an indicator for something and the speed was the indicator? Because I thought it would maybe be quality, like smoothness, but then I was thinking at the back of my head the only thing the watch is going to be able to measure is my heart rate all the time. So it was a bit like I didn’t know what it was for.” (9234, M, 60-69, 2 conditions)

The majority of participants reported that the tasks were too easy, making them feel ‘pointless’. A few thought the aim of the active tasks was to affect biological reactions such as heart rate or blood pressure that the watch could then track, but they felt that the tasks were too easy to produce meaningful results:

“Only as thinking, well, my pulse hasn’t changed so what else are you going to record from it.” (4987, M, 50-59, 2 conditions)

For those who did have difficulty with physical activities or who experience high levels of pain, they reported that the tasks were useful. Finding the right balance of active tasks that suits all levels of ability is complex and as with the symptom questions, the suggestion was made that it would be of more benefit if at onboarding the tasks could be tailored to a person’s ability.

### Limitations of the interactivity of the watch

At enrolment participants were told that the watch would only have access to the WYS app and would not have access to other apps. They were also advised that they would not be able to see the results of either their symptom monitoring questions or the tracking data (data collected in the background) until the end of the study. Despite this information, the lack of feedback was seen as a major barrier for some in terms of engaging participants over a longer period:

“So, I thought…not disappointed but I thought that even though it's a trial and a feasibility study, whatever, there could have been a bit more information gleaned from it, if you like.” (4489, M, 50-59, 1 condition)

Another theme that arose was desire for the watch to become more than a symptom tracker but actually support self-management (e.g. alerting someone to take their medication if their BP gets too high or too low, or to move if they have been sedentary for an extended period):

“And basically, you can do exactly the same with the watch. If the watch spots that somebody’s heart rate is going like the clappers, again, it can actually say, well hang on, are you doing exercise? No. Right, well sit down, calm down, slow down. And works the opposite if it goes too slow… But I do believe that with modern technology these days, symptoms like that can be spotted at an earlier stage and dealt with better.” (3545, M, 40-49, 2 conditions)

Another participant suggested that this would not even need to be provided as real-time feedback, a summary that could be discussed later would be useful:

“When I go and get my blood pressure monitor, I’ve got to physically remember whereas if I had it on the watch and it monitored, you know, it gave you a monthly review of…I could go to the doctors then and just say, well here’s me, this is what it is on a regular basis and it could’ve took it for me. I think that would’ve been a very big help because I have been having high blood pressure and I’m, you know, wanting to try for a baby in the near future.” (5501, F, 30-39, 2 conditions)

Some participants reflected that they would like to be able to see how their conditions interacted with each other in real-time:

“So you can actually, in one respect you can use the data to help manage things because I know that I’m always stiff pretty much all day round but especially in the mornings and in the evening. Now I could actually look at different, my heart rate or my temperature and see if they correlate and correspond with when I am stiff, when I am in pain and that as well.” (6655, M, 40-49, 3 conditions)

In general, users seemed to have fairly high expectations of the technology. It is important to meet those expectations in an increasingly digitally literate population, in order to increase long-term engagement.

#### Sub-group comparison

We wanted to see if there were any differences caused by age or number of LTCs. To explore this, participants were split into age groups <60 years (n=13) vs ≥60 years (n=7), and <3 LTCs (n=15) vs ≥3 LTCs(n=5), to see if any of these factors influenced engagement or experience.

### Impact of age on engagement

Older people (≥60 years of age) engaged well with the system and were able to complete the questions and active tasks. According to participant narratives there were no noticeable differences in ability to use the watch or complete the tasks by age. Similar numbers in each age group found the active tasks ‘too easy’ (as above). No participants expressed that they specifically found age to be a barrier for using the app.

#### Impact of MLTC-M on engagement

Looking at whether the number of LTCs (3 or more) impacted engagement, there were no clear differences either in levels of engagement or in the views expressed by participants.

In general participants did not seem to consider the management of more than one LTC to be a factor in either engagement or use of the watch. In fact, only a small number of participants expressed interest in how the different LTCs interacted with each other, mainly focussing on a single condition that tended to impact their life more significantly than other conditions they had. The only condition that was raised by participants as a barrier to engagement was the presence of severe or chronic pain.

For some participants, the pain they experienced impacted how and when they could use the watch. This was particularly an issue for the physical activities but in one case even touching the watch during an episode of severe pain was too much.

When a particular participant was asked why they had not completed any of the walking activity tasks, they responded:

‘Yes, because it was the pain, a lot of that is because of the pain that I’d been in. You have got to catch me at the right time of day, in order for me to do something like that. And it always caught me when I’m in a lot of pain. So if I am in a lot of pain I can't physically actually get up and walk, do walking like that. I have just got to sit down and rest as much as possible.” (6655, M, 40-49, 3 conditions)

Another participant pointed out that pain also interfered with their ability to complete the tasks within the response window. Another participant with severe arthritis in their wrists described how wearing the watch caused irritation by rubbing on the joint. However, even though they experienced some discomfort they said that they still enjoyed the process, and that generally it did not impact responding to the question prompts.

## Discussion

### Summary

The key points that this qualitative analysis raised were around: motivations for participant engagement, need for personalisation, and clearer communication about the purpose of the study. The key finding was that there was a lack of evidence that MLTC-Ms cause problems of engagement compared to other studies that focus on single conditions, or the expectation that it would be harder to collect PGHD in this population.

### Comparison to other studies

In a review of barriers and facilitators to engagement with remote measurement technologies for health, the authors reported 33 studies using a variety of methodologies.^14^ The article, highlighted a number of gaps in the design of the studies particularly around quantitative assessment of usage and acceptability of the technology. In the current study we have focussed on both the usage (usage data has been presented elsewhere)^12^ and views on acceptability of the WYS app. However, we acknowledge that the three-month data collection period for the wearable device may not be sustained over a longer period, particularly considering the lack of ‘real time' feedback that participants wanted.

The review by Simblett et al^14^ identified a number of barriers and facilitators related to: health status, perceived utility and value, motivation, convenience and accessibility, and usability across studies. Our results were broadly in line with these and therefore with studies outside MLTC-M. However, our results suggest that the presence of severe or chronic pain and the importance of tailoring may be issues that require greater focus in MLTC-M.

In another study exploring perspectives around a hypothetical smartphone app for depression, both patients and physicians had high expectations for app content.^15^ The authors reported that the key expectations were around ease of use and personalised content. In the results we present here, participants were content with the usability of the wearable device. However, even though questions and tasks were specific to organ systems, participants still expressed a desire for the ability to further tailor the questions to their specific symptoms and to adjust the levels of the physical tasks to suit their current abilities.

Our previous studies^7,16^ have shown that it is possible to collect PGHD from patients with LTC.^17^ This qualitative study explored acceptability of collecting daily PGHD for three months using the smartwatch app in a population of older people with MLTC-M. We found that they were willing and able to collect data, and we identified barriers to and facilitators of ongoing engagement.

### Strengths and limitations

The WYS app is the first study we are aware of that has developed a symptom data collection system for people with MLTC-M. Previous studies have focussed on disease-specific questions in younger, healthier populations.^18^ All participants were volunteers and were reimbursed for participation. Whilst a wide range of age groups took part in this study, the age distribution was younger than in MLTC-M populations that are not self-selecting.^19^ There is clearly a need to continue to test engagement in more diverse groups, but the study provides a useful first test of PGHD in MLTC-M. Due to the timing of this research, when covid restrictions were still in place and funding/time limitations, no further work was done to test the rigor of the interpretation of this data.

### Meaning of the study and implications

Overall, participants reported that the app was easy to use and that the number of alerts and questions was not a barrier to engagement. Our research around the completion of PGHD has increased from initial studies of weekly collection to once daily,^16^ through six items per day^20^ and now to 20 plus items per day^12^ in MLTC without participants expressing any major problems. Most participants reported satisfaction with the scope of the data being collected. The repetitive nature of the data collection led to some irritation, and there were concerns over the active tasks being too easy to complete. An option may be to vary the order questions are asked or adapting the frequency of some questions, for example if serial ‘0’s are entered. Some participants highlighted issues in their understanding about the purpose of the study which suggest that further attention to research materials and onboarding was required, especially when there are differences between what is important to the participant and the researcher, and when the utility of tasks might not be so apparent to patients. Participants highlighted a number of benefits for them - understanding how their symptoms change through time (which highlighted the lack of feedback); understanding correlations or triggers for flares; allowing interventions to be guided by PGHD (including ‘just in time’ interventions); personalising the technology; and supporting better clinical care through sharing with professionals.

A number of participants highlighted the need for greater flexibility and personalisation. For example, without the ability to add qualifying comments, they reported that the app was not capturing the full picture of their symptoms. This may be a result of participants having more than one LTC, as some symptoms could relate to a number of conditions or be located in different parts of the body, and some participants felt it important to differentiate between these. Similarly, allowing people to select active tasks might ensure they were more relevant to their abilities, and more work is needed to understand how best to set up condition-specific questions at baseline.

Almost all participants were able to complete the three month data collection period, and around half suggested a longer period of at least 12-18 months would be beneficial for them to try and track changes over time. However, they suggested that long-term engagement would require more interactivity or feedback from the technology. This is supported by previous studies which likewise showed incorporating a mechanism for feedback to users would be preferable.^21,22^

There were no substantive differences in the way participants with more LTCs engaged with the system as compared to those with fewer conditions, and older participants were just as capable of using the system and actually chose to engage more fully. Rather than MLTC-M being a barrier to engagement, some participants report focussing on a single condition that tended to impact their life more significantly, consistent with previous research.^23^ The only condition that raised as a barrier to engagement was the presence of severe or chronic pain.

## Conclusion

The Academy of Medical Sciences report identified exploration of the use of consumer technology to track and monitor symptoms of people living with MLTC-M. Our study is the first to provide preliminary data to show that such use is feasible and acceptable, and has highlighted factors (such as the need for personalisation and feedback) that may enhance its use among patients with MLTC-M.

## ORCID iDs

Cassandra Kenning https://orcid.org/0000-0001-6041-4051

Peter Bower https://orcid.org/0000-0001-9558-3349

Nicola Small https://orcid.org/0000-0002-7879-7967

Syed Mustafa Ali https://orcid.org/0000-0001-9393-9049

Benjamin Brown https://orcid.org/0000-0001-9975-4782

Brian McMillan https://orcid.org/0000-0002-0683-3877

Caroline Sanders https://orcid.org/0000-0002-0539-928X

Sabine N Van der Veer https://orcid.org/0000-0003-0929-436X

William G Dixon https://orcid.org/0000-0001-5881-4857

John McBeth https://orcid.org/0000-0001-7047-2183

## References

[1] Department of Health . Long Term Conditions Compendium of Information: Third edition. 2012 (Accessed 07/08/2020).

[2] National Health Service . NHS Five Year Forward View. 2014. (Accessed 03/08/2020).

[3] Barnett K Mercer SW Norbury M , et al. Epidemiology of multimorbidity and implications for health care, research, and medical education: a cross-sectional study. Lancet*.* 2012; 380: 37-43. 22579043 10.1016/S0140-6736(12)60240-2

[4] Cottrell E Yardley S . Lived experiences of multimorbidity: An interpretative meta-synthesis of patients', general practitioners' and trainees' perceptions. Chronic Illn*.* 2015 Dec;11(4):279-303. doi: 10.1177/1742395315574764. Epub 2015 Mar 12. PMID: 25770097. 25770097

[5] Boye LK Mogensen CB Mechlenborg T, et al. Older multimorbid patients’ experiences on integration of services: a systematic review. BMC Health Serv Res 19, 795 (2019). 31690308 10.1186/s12913-019-4644-6 PMC6833141

[6] The Academy of Medical Sciences . Multimorbidity: a priority for global health research. Academy of Medical Sciences. 2018.

[7] Dixon WG Michaud K . Using technology to support clinical care and research in rheumatoid arthritis. Curr Opin Rheumatol 2018;30:27681. 10.1097/BOR.0000000000000485 PMC5895111 29369089

[8] IQVIA Institute for Human Data Science . The Growing Value of Digital Health. 2017. https://www.iqvia.com/engb/institute/reports/the-growing-value-of-digital-health (date last accessed 2 June 2019).

[9] Corbett J D’Angelo C Gangitano L , et al. Future of health: findings from a survey of stakeholders on the future of health and healthcare in England. Rand Health 2017;7:1. 10.7249/rhq-07-03-01 PMC5873518 29607245

[10] Morton K Dennison L May C, et al. Using digital interventions for self-management of chronic physical health conditions: a meta-ethnography review of published studies. Patient Educ Couns 2017;100:61635. 10.1016/j.pec.2016.10.019 PMC5380218 28029572

[11] Beukenhorst A Howells K Cook L , et al. Engagement and Participant Experiences with Consumer Smartwatches for Health Research: a Longitudinal, Observational Feasibility Study. J Med Internet Res 2020;8(1):e14368. 10.2196/14368 PMC7016619 32012078

[12] Ali SM Selby DA Kazi K , et al. Engagement with consumer smartwatches for tracking symptoms of individuals living with multiple long-term conditions (multimorbidity): a longitudinal observational study. J Multimorb Comorb 2021;11:1-13. 10.1177/26335565211062791 PMC8637784 34869047

[13] QSR International (1999) NVivo Qualitative Data Analysis Software v12 [Software].

[14] Simblett S Greer B Matcham F , et al. Barriers to and Facilitators of Engagement With Remote Measurement Technology for Managing Health: Systematic Review and Content Analysis of Findings. J Med Internet Res 2018;20(7):e10480. 30001997 10.2196/10480 PMC6062692

[15] Patoz MC Hidalgo-Mazzei D Blanc O , et al. Patient and physician perspectives of a smartphone application for depression: a qualitative study. BMC Psychiatry. 2021 Jan 29;21(1):65. 33514333 10.1186/s12888-021-03064-x PMC7847000

[16] Austin L Sharp C A van der Veer S N , et al. Providing ‘the bigger picture’: benefits and feasibility of integrating remote monitoring from smartphones into the electronic health record: Findings from the Remote Monitoring of Rheumatoid Arthritis (REMORA) study. Rheumatology 2020;59:367378. 10.1093/rheumatology/kez207 PMC7223265 31335942

[17] Gandrup J Ali S M McBeth J , et al. Remote symptom monitoring integrated into electronic health records: A systematic review. JAMIA 2020;11:1752-1763. 10.1093/jamia/ocaa177 PMC7671621 32968785

[18] Cornet VP Holden RJ . Systematic review of smartphone-based passive sensing for health and wellbeing. J Biomed Inform [Internet]. 2018 Jan;77:120-132. 29248628 10.1016/j.jbi.2017.12.008 PMC5793918

[19] Wang J Wang Y Wei C , et al. Smartphone Interventions for Long-Term Health Management of Chronic Diseases: An Integrative Review. Telemed e-Health [Internet]. 2014 Jun;20(6):570-583. 10.1089/tmj.2013.0243 24787747

[20] Vivekanantham A Selby D Lunt M , et al. Day-to-day variability of knee pain and the relationship with physical activity in people with knee osteoarthritis: an observational, feasibility study using consumer smartwatches. BMJ Open 2023;13:e062801. doi:10.1136/bmjopen-2022-062801 PMC10016308 36914192

[21] Druce KL Dixon WG McBeth J . Maximizing Engagement in Mobile Health Studies. Rheum Dis Clin North Am [Internet]. 2019 May;45(2):159–172. 30952390 10.1016/j.rdc.2019.01.004 PMC6483978

[22] Fischer F Kleen S . Possibilities, Problems, and Perspectives of Data Collection by Mobile Apps in Longitudinal Epidemiological Studies: Scoping Review. J Med Internet Res [Internet]. 2021 Jan 22;23(1):e17691. Available from: https://www.jmir.org/2021/1/e17691/33480850 10.2196/17691 PMC7864774

[23] Sathanapally H Sidhu M Fahami R , et al. Priorities of patients with multimorbidity and of clinicians regarding treatment and health outcomes: a systematic mixed studies review. BMJ Open 2020;10:e033445. 10.1136/bmjopen-2019-033445 PMC7045037 32051314

